# Molecular Mechanisms of Aggregation of Canine SOD1 E40K Amyloidogenic Mutant Protein

**DOI:** 10.3390/molecules28010156

**Published:** 2022-12-24

**Authors:** Kento Wakayama, Shintaro Kimura, Yui Kobatake, Hiroaki Kamishina, Naohito Nishii, Satoshi Takashima, Ryo Honda, Yuji O. Kamatari

**Affiliations:** 1 Faculty of Applied Biological Sciences, Gifu University, 1-1 Yanagido, Gifu 501-1193, Japan; 2 Life Science Research Center, Gifu University, 1-1 Yanagido, Gifu 501-1193, Japan; 3 Neuroprotection Research Laboratory, Departments of Radiology and Neurology, Massachusetts General Hospital and Harvard Medical School, Boston, MA 02129, USA; 4 Kyoto AR, 33 Sayama-Nakamichi, Kumiyama, Kuze, Kyoto 613-0036, Japan; 5 The United Graduate School of Veterinary Sciences, Gifu University, 1-1 Yanagido, Gifu 501-1193, Japan; 6 United Graduate School of Drug Discovery and Medical Information Sciences, Gifu University, 1-1 Yanagido, Gifu 501-1193, Japan; 7 Institute for Glyco-Core Research (iGCORE), Gifu University, 1-1 Yanagido, Gifu 501-1193, Japan

**Keywords:** amyotrophic lateral sclerosis (ALS), degenerative myelopathy, superoxide dismutase 1, protein aggregation, amyloidogenic proteins

## Abstract

Canine degenerative myelopathy (DM) is a human amyotrophic lateral sclerosis (ALS)-like neurodegenerative disease. It is a unique, naturally occurring animal model of human ALS. Canine DM is associated with the aggregation of canine superoxide dismutase 1 (cSOD1), which is similar to human ALS. Almost 100% of cases in dogs are familial, and the E40K mutation in cSOD1 is a major causative mutation of DM. Therefore, it is important to understand the molecular mechanisms underlying cSOD1(E40K) aggregation. To address this, we first analyzed the structural model of wild type cSOD1. Interactions were evident between amino acid E40 and K91. Therefore, the mutation at residue E40 causes loss of the interaction and may destabilize the native structure of cSOD1. Differential scanning fluorimetry revealed that the E40K mutant was less stable than the wild type. Moreover, stability could be recovered by the E40K and K91E double mutation. Acceleration of amyloid fibril formation in vitro and aggregate formation in cells of cSOD1(E40K) was also suppressed by the introduction of this double mutation in thioflavin T fluorescence assay results and in transfectant cells, respectively. These results clearly show the importance of the interaction between amino acid residues E40 and K91 in cSOD1 for the stability of the native structure and aggregation.

## 1. Introduction

Amyotrophic lateral sclerosis (ALS) is a progressive and fatal neurodegenerative disease associated with aggregation of superoxide dismutase 1 (SOD1) protein [1,2,3]. In dogs, canine degenerative myelopathy (DM) is a neurodegenerative disease associated with aggregation of canine SOD1 (cSOD1), as occurs in ALS, and affects many dog breeds [4,5]. The clinical signs of DM include pelvic general proprioceptive ataxia and upper motor neuron paraparesis, which progress to lower motor neuron tetraplegia. DM-affected dogs eventually die from respiratory failure, approximately three years or more after disease onset [6,7,8]. Because of the similar clinical signs, neuropathological findings, and involvement of SOD1, DM can be considered a unique naturally occurring animal model for human ALS [7,9,10].

Dogs with DM have recently been found to carry mutations in SOD1. Resequencing of SOD1 in normal and DM-affected dogs revealed the c.118G >A missense mutation in exon 2, which predicted an E40K substitution [4]. In humans, approximately 10% of cases are considered familial ALS and more than 170 mutations in SOD1 have been identified [11,12,13,14]. In contrast, 100% of cases are considered familial in dogs with DM, and the E40K is a major causative mutation of DM [15]. The T18S mutation in cSOD1 has also been reported as another causative mutation for DM, but it is present only in the Bernese Mountain Dog [16].

Human SOD1 (hSOD1) is a ubiquitously expressed antioxidant enzyme involved in the deactivation of toxic superoxide radicals. It is a homodimer protein of two 153 amino acid subunits [17,18]. The native structure of the protein contains eight β-strands, catalytic copper ion, structurally important zinc ion, electrostatic loop element that forms a portion of the active site funnel, and intramolecular disulfide bond between cysteine 57 and cysteine 146 [19,20,21]. A structure of cSOD1 expected to be similar to that of hSOD1 because of 80% sequence identity between dog and human [22]. Since the E40K mutation is a leading cause of DM development, it is important to understand the molecular mechanisms of aggregation of the E40K mutant protein of cSOD1. To address this, we first analyzed the structural model of wild type cSOD1 [cSOD1(WT)] and found interactions between the amino acid E40 and K91. Therefore, mutation at residue E40 may result in the loss of its interaction and destabilization of the native structure of cSOD1.

We have previously reported that the E40K mutation did not alter the thermal stability of the secondary structure of cSOD1, as monitored by far-ultraviolet (UV) circular dichroism (CD) spectroscopy [23]. However, it is known that a compact denature state, molten globule (MG) state, accumulate during the folding of some proteins [24,25,26]. Proteins in the MG state are more or less compact (hence, “globule”), but lack the specific tight packing of amino acid residues that creates the rigid tertiary structure of completely folded proteins (hence, “molten”). This intermediate state may also accumulate during the unfolding of cSOD1. Therefore, it is important to investigate unfolding of the tertiary structure of cSOD1. In this study, we employed differential scanning fluorimetry (DSF), which permitted monitoring of the unfolding of the specific tight packing of the hydrophobic core of cSOD1. We found that the E40K mutant was less stable than the WT. Moreover, stability could be recovered by the E40K and K91E double mutation. These results clearly show the importance of the interaction between amino acid residues E40 and K91 in cSOD1.

## 2. Results

### 2.1. Structural Analysis of cSOD1 Proteins

**Figure 1:**
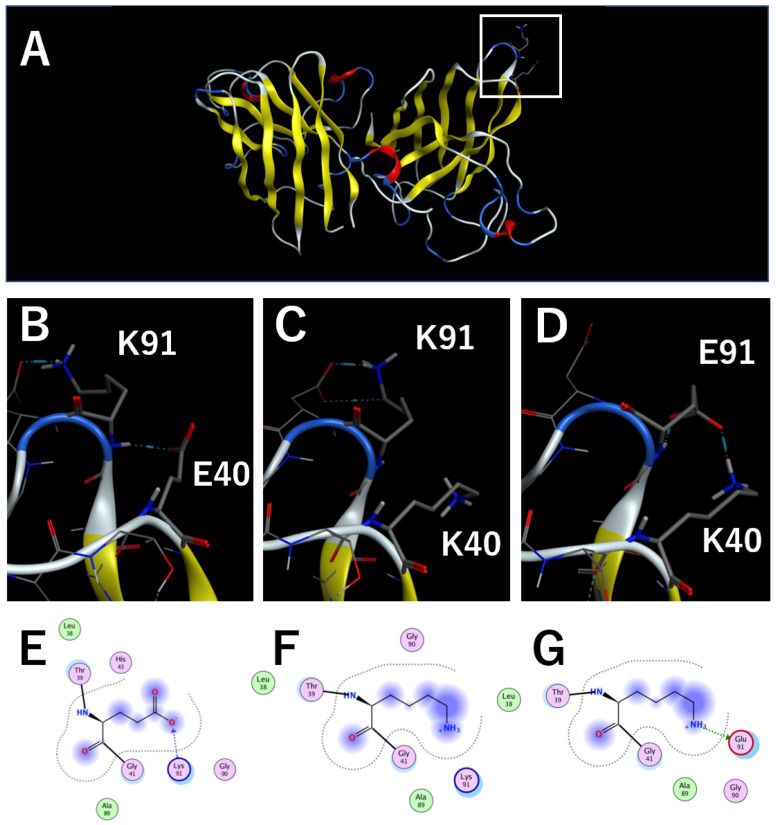
Homology models of cSOD1 WT and mutants. (**A**). The entire cSOD1(WT) dimer structure. α-helix, β-sheet, and turn structures are colored red, yellow, and blue, respectively. (**B**~**D**). Close-up displays of the region around residue 40 of cSOD1(WT) (**B**), (E40K) (**C**), and (E40K,K91E) (**D**) shown in the white square in Figure 1A. The locations of E40 and K91 are noted in one of the two subunits visible in the image. The interaction between residues E40 and K91 are shown by blue dotted lines. E~G. MOE interaction map of residue 40 of cSOD1(WT) (**E**), (E40K) (**F**), and (E40K,K91E) (**G**).

To understand the interaction in the native structure of cSOD1, structures of cSOD1(WT) and mutants were created using the homology modeling function of the Molecular Operating Environment (MOE) software package (Chemical Computing Group, Montreal, Quebec, Canada). Figure 1A shows the entire cSOD1 (WT) dimer structure. Figure 1B provides a close-up view of the region around residue E40. E40 is close to K91, and there is a hydrogen bond between the side chain of E40 and the main chain NH of K91 (Figure 1B,E). The interaction between residues 40 and 91 was absent in cSOD1(E40K) (Figure 1C,F). Interestingly, this interaction was recovered in the cSOD1(E40K,K91E) double mutant by forming hydrogen bond and ionic interactions between the side chain of K40 and the side chain of E91 (Figure 1D,G). From the structural analysis of the cSOD1 protein, we expected that the interaction between residues 40 and 91 may contribute to stabilizing the native structure of cSOD1(WT). Stability difference of amino acid substitution was also calculated using Residue Scan function of MOE. Stability difference from WT to the E40K mutant was 1.56 kcal/mol, which indicated that cSOD1(E40K) is significantly unstable compared to cSOD1(WT). In contrast, that from WT to the E40K and K91E double mutant was only 0.30 kcal/mol, which indicated that stability of cSOD1(E40K,K91E) is similar to that of cSOD1(WT).

### 2.2. DSF Evaluation of Structural Stability of cSOD1 WT and Mutants

**Figure 2:**
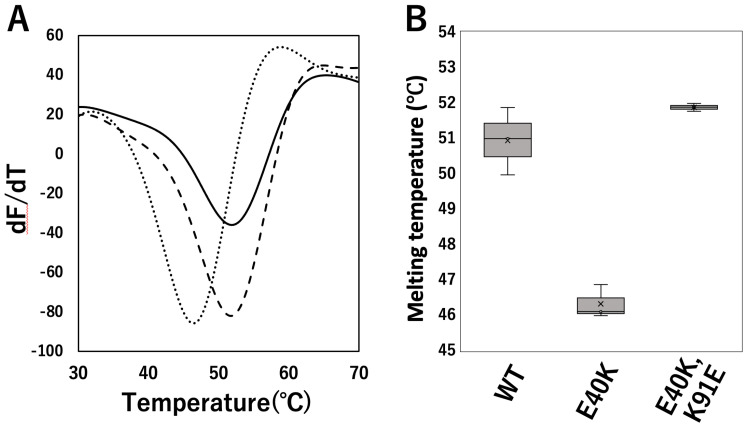
Thermal unfolding of cSOD1 WT and mutants monitored by differential scanning fluorimetry (DSF). (**A**) DSF curves of cSOD1(WT) (solid line), cSOD1(E40K) (dotted line), and cSOD1(E40K,K91E) (dashed line). (**B**) Comparison of the melting temperature (*T*_m_) of cSOD1(WT) and mutants.

Next, we experimentally evaluated the structural stability of cSOD1 mutants. We produced recombinant cSOD1(WT), (E40K), and (E40K,K91E) and observed the thermal unfolding of cSOD1s using DSF (Figure 2). The melting temperatures (*T*_m_) of the tertiary structures obtained by DSF are summarized in Table 1. The *T*_m_ value of the cSOD1(E40K) mutant was lower than that of cSOD1(WT) (*p* = 0.0002), indicating that the tertiary structure of cSOD1(E40K) is less stable than that of cSOD1(WT). In contrast, the *T*_m_ value of cSOD1(E40K,K91E) was higher than that of cSOD1(E40K) (*p* < 0.0001) and was almost the same as that of cSOD1(WT). This indicated that the stability was recovered by the double mutation, E40K and K91E.

### 2.3. Thioflavin T (ThT) Fluorescence Assay Evaluation of Amyloid Fibril Formation of cSOD1 WT and Mutants

**Figure 3:**
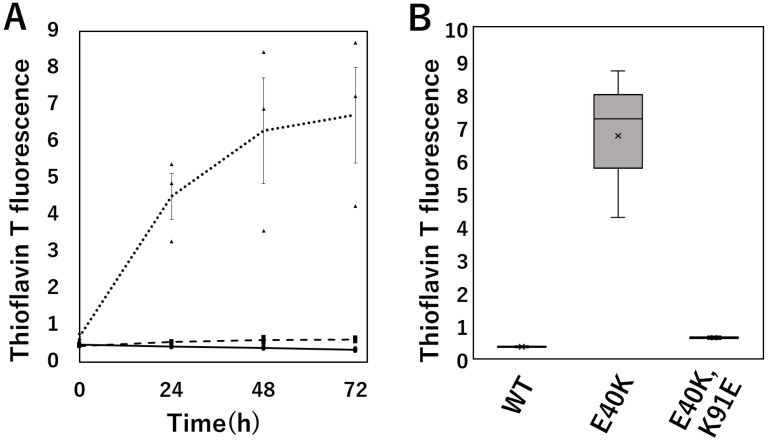
Amyloid fibril formation by cSOD1 WT and mutants monitored by the thioflavin T fluorescence assay. (**A**). Time courses of amyloid fibril formation of cSOD1(WT) (solid line), cSOD1(E40K) (dotted line), and cSOD1(E40K,K91E) (dashed line). (**B**). Plot of the fluorescence intensity of the various cSOD1s at 72 h.

ThT selectively binds to amyloid fibrils and increases fluorescence intensity. We performed a ThT assay to monitor the kinetics of amyloid fibril formation by cSOD1s (Figure 3). Structure of the amyloid fibrils formed in this condition was observed by transmission electron microscopy and was worm-like fibrils [23,27].

cSOD1(E40K) significantly increased the kinetics of amyloid fibril formation compared to WT (*p* = 0.0024). In contrast, cSOD1(E40K,K91E) mutations decreased the kinetics of amyloid fibril formation compared to that of cSOD1(E40K) (*p* = 0.003) and was almost the same as that of cSOD1(WT).

### 2.4. Evaluation of Aggregate Formation of GFP-Tagged cSOD1(WT) and Mutants in Transfectants

**Figure 4:**
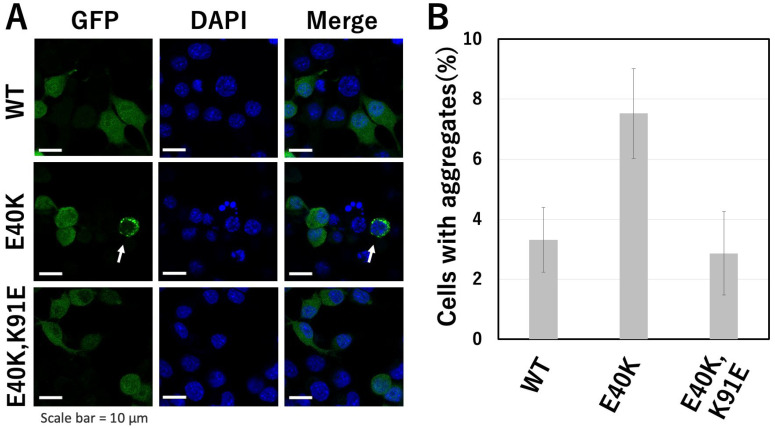
The aggregate formation in transfectants of GFP-tagged cSOD1s. (**A**) Confocal micrographs of GFP-tagged cSOD1. Aggregates, which were observed as bright spots, were shown by a white arrow. (**B**) The proportion of cells containing cSOD1 aggregates.

To evaluate aggregate formation by cSOD1 in cells, we examined cSOD1-transfected cells and counted the number of cells that contained cSOD1 aggregates evident as bright spots in the cytoplasm of Neuro2a cells (Figure 4). Neuro2a cells are mouse neuroblasts isolated from brain tissue. It is a one of the most popular neuronal cells and we have been using this cell to evaluation of aggregate formation of SOD1 [5,23,27]. All the cSOD1s-transfected cells formed aggregates. However, the proportion of cells containing the aggregates in green fluorescent protein (GFP)-tagged cSOD1(E40K)-transfected cells was significantly higher (mean ± standard error, 7.5 ± 1.5) than in GFP-tagged cSOD1(WT)-transfected cells (3.3 ± 1.1) (*p* = 0.0003). The proportion of cells containing cSOD1 aggregates in GFP-tagged cSOD1(E40K,K91E)-transfected cells was significantly lower (2.9 ± 1.4) than that in GFP-tagged cSOD1(E40K)-transfected cells (*p* = 0.0002) and similar to that in GFP-tagged cSOD1(WT)-transfected cells.

## 3. Discussion

### 3.1. Denaturation and Aggregation of cSOD1

Canine DM is a human ALS-like neurodegenerative disease. It is associated with the aggregation of cSOD1, similar to that in human ALS. In dogs, almost 100% of the cases are familial, and E40K is a major causative mutation of DM. Therefore, it is important to understand the molecular mechanisms underlying cSOD1(E40K) aggregation.

**Figure 5:**
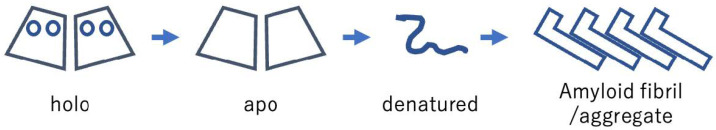
Schematic representation of denaturation and aggregation of cSOD1.

SOD1 can adopt both Cu^2+^/Zn^2+^-unbound (apo) and -bound (holo) states. The structural stability of holo-SOD1 is significantly higher than that of apo-SOD1 [23,28]. Then, apo-SOD1, but not holo-SOD1, resulted in fibrous aggregates in the presence of pathogenic mutations. Therefore, we used apo-cSOD1s to evaluate the structural stability and amyloidogenic propensity in this study (Figure 5).

### 3.2. The E40K Mutant Is Less Stable and Displays Higher Amyloidogenic Propensity In Vitro and in Cells

Structural analysis of the cSOD1 proteins indicated that the interaction between E40 and K91 in the WT structure was lost in the cSOD1(E40K) structure (Figure 1). As expected from the structural analysis, cSOD1(E40K) protein was significantly unstable compared to the WT protein, as shown by DSF (Figure 2). The lower stability of cSOD1(E40K) indicates a higher population of denatured (partially or fully unfolded) cSOD1(E40K) compared to that of cSOD1(WT). The denatured conformers are important for aggregate formation (Figure 5). Because protein molecules in solution exist as an equilibrium of different conformers, the denatured conformers exist in low but definite populations even under physiological conditions. The higher population of the denatured state of cSOD1(E40K) is expected to accelerate amyloid fibril formation. Acceleration of amyloid fibril formation in vitro (Figure 3) and aggregate formation of cSOD1(E40K) in cells (Figure 4) were observed.

### 3.3. The E40K and K91E Double Mutant Recover Stability and Lessen Amyloidogenic Propensity In Vitro and in Cells

Structural analysis of the E40K and K91E double mutant indicated an interaction between K40 and E91 (Figure 1D,G), which was lost in the cSOD1(E40K) structure. As expected from the structural analysis, the stability could be recovered by the double mutation (Figure 2). Moreover, the acceleration of amyloid fibril formation in vitro and aggregate formation of cSOD1(E40K) in cells was also suppressed by the double mutation shown by the ThT fluorescence assay (Figure 3) and transfectant cells (Figure 4), respectively. These results clearly demonstrate the importance of the interaction between amino acid residues E40 and K91 in cSOD1.

### 3.4. Importance of the Interaction between Amino Acid Residues E40 and K91 in cSOD1

**Figure 6:**
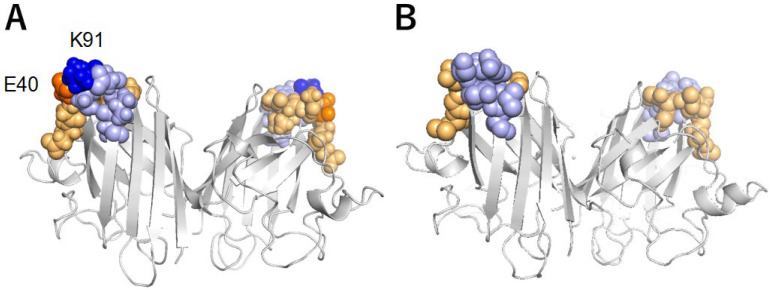
“β-barrel plug” in canine (**A**) and human (**B**) SOD1 structures. The residues 37–42 and 90–95 comprising the β-barrel plug are colored light orange and blue in the SOD1 structures, respectively. E40 and K91 are colored in bright orange and blue in cSOD1, respectively.

Loops 37–43 and 90–95 are located on one end of the β-barrel of SOD1 (Figure 6), where amino acid residues 37–43, 90–95, and 144 comprise the multiple interaction networks [29]. This region is termed the “β-barrel plug” and is proposed to play an important role in the structural stabilization of SOD1 by protecting edge strands and hydrophobic packing that are believed to be aggregation initiation sites [29,30,31,32]. Familial ALS mutations related to the β-barrel plug region, such as G93A and G37R, have been reported in humans [29,33]. Actually, the G37R mutation of human SOD1 was reported to be unstable compared to WT [29,34]. The E40 residue is in loop 37–43 and residue K91 is in loop 90–95 in the canine SOD1 structure (Figure 6A). Our findings are consistent with this idea, and demonstrate the importance of the β-barrel plug.

In addition to the lower stability of cSOD1(E40K), decreased negative charge of the E40K mutation contributes to increasing amyloidogenic propensity. Previously, we reported that cSOD1(WT) has a negative net charge (−4.5) at physiological pH and that the E40K mutation decreases the negative net charge to −2.0 [23]. A decreased net charge promotes protein aggregation by enhancing the intermolecular electrostatic forces between unfolded polypeptides [35,36]. In contrast, the cSOD1(E40K,K91E) did not exhibit a change in the negative net charge. Thus, the E40K and K91E mutation may suppress aggregate formation. Both factors, the lower stability of cSOD1(E40K) shown in this study and decreases the negative net charge shown in previous study, contribute to higher aggregation propensity of cSOD1(E40K).

### 3.5. Molecular Evolution and Equine SOD1

**Figure 7:**
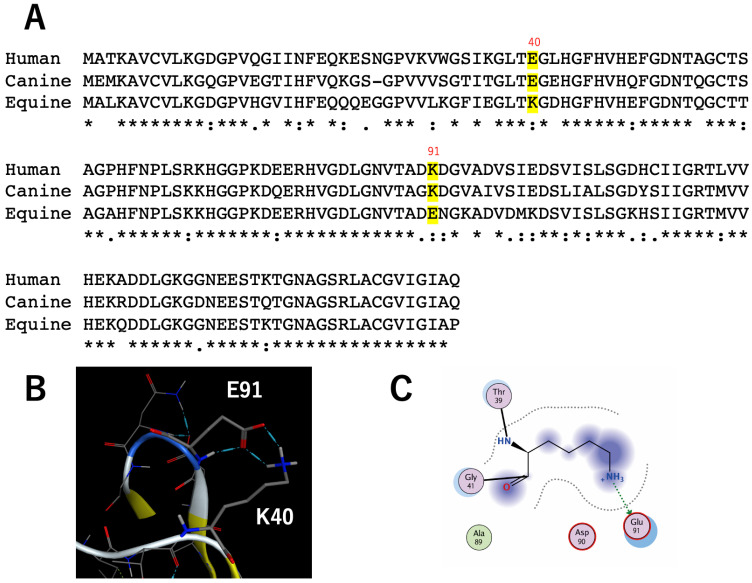
(**A**). Sequential alignment of human, canine, and equine SOD1s. (**B**). Close-up displays of the region around residue 40 of equine SOD1 homology model. The locations of K40 and E91 are noted. The interactions are shown by blue dotted lines. (**C**). MOE interaction map of residue 40 of the equine SOD1 homology model.

The E40 residue is highly conserved in mammals, except horses, donkeys, and armadillos [37]. Mutation at E40 cause DM in dogs, but DM has not been reported in horses. The reason why K40 was selected for equine SOD1 is unknown. Figure 7 shows the sequential alignments of human, canine, and equine SOD1s. Interestingly, residue 91 of equine SOD1 is glutamate (E), which is the same combination as cSOD1(E40K,K91E) in this report. In this study, we showed that cSOD1(E40K,K91E) is stable and less amyloidogenic. This can be true for equine SOD1, which contains K40 and E91. We created a structure model of equine SOD1 using the homology modeling function of MOE. In the equine SOD1 structure, there were hydrogen bond and ionic interactions between the side chain of K40 and the side chain of E91 (Figure 7B,C).

This combination, instead of E40 and K91, can also stabilize the β-barrel of SOD1 as the β-barrel plug, and have been selected evolutionarily for horses, donkeys, and armadillos.

## 4. Materials and Methods

### 4.1. Computational Analysis of cSOD1 Structures

Homology modeling and structural analysis of cSOD1(WT) and mutants were performed using the MOE software package. The cSOD1 sequences and crystal structure coordinates of the human SOD1 [Protein Data Bank code: 3ECU] [38] were loaded into the MOE. The sequences of canine and human SOD1 were aligned. Ten models were independently constructed using MOE. The best models were selected for full energy minimization and further inspection. The models were analyzed using the protein geometry function by MOE. There were no unacceptable deviations in the model and no outliers in the Ramachandran plot. Stability difference of amino acid substitution was calculated using Residue Scan function of MOE.

### 4.2. Preparation of Recombinant Proteins

Bacterial expression plasmid vectors, pGEX4T-1 (Cytiva, Tokyo, Japan), for the production of canine recombinant cSOD1 proteins were transformed into competent (BL21) *Escherichia coli*. Protein expression and purification were performed as previously described [5,23]. The purity of the recombinant proteins was >95%, as confirmed by SDS-PAGE. The protein concentration was determined based on the absorption at 280 nm using an UV spectrometer (Shimadzu, Kyoto, Japan).

### 4.3. DSF

The structural stability of cSOD1s was examined using DFS. For experiments using apo-cSOD1, recombinant cSOD1 proteins were diluted to 100 µM with 40 mM sodium phosphate (pH 7.4). EDTA (10 mM) and SYPRO Orange (Thermo Fisher Scientific, Waltham, MA, USA) were added to the solutions. DFS experiments were carried out using StepOnePlus RT-PCR (Applied Biosystems, Waltham, MA, USA) with temperature ramp of 1 °C per min between 25 °C and 95 °C. This experiment was repeated three times.

### 4.4. ThT Fluorescence Assay to Monitor Amyloid Formation

The ThT fluorescence assay was used to monitor amyloid formation by apo-cSOD1 proteins. Solutions containing 40 µM apo-cSOD1, 10.5 mM EDTA, and 10 µM ThT were prepared in each well of a 96-well plate. Solutions in the plate were later shaken using a plate shaker (TAITEC, Saitama, Japan) at a rate of 600 rpm at 37 °C. The formation of the amyloid fibril assembly was monitored through the increase in ThT fluorescence at 495–505 nm (excitation wavelength of 405 nm) every 24 h using a VARIOSKAN FLASH multi-spectrum microplate reader (Thermo Fisher Scientific). Fluorescence at 72 h was standardized to that of the dimethylsulfoxide control group. The experiments were repeated three times.

### 4.5. Aggregate Formation in cSOD1 Transfectants

Neuro2a mouse neuroblastoma cells were dispensed in wells of an 8-well chamber slide (6.0 × 10^4^ cells/well) in Dulbecco’s modified Eagle’s medium (DMEM; FUJIFILM Wako, Osaka, Japan) supplemented with 10% fetal bovine serum (FBS; Biosera, Kansas City, MO, USA). Plasmids of GFP-tagged cSOD1(WT), GFP-tagged cSOD1(E40K), or GFP-tagged cSOD1(E40K,K91E) (0.4 μg/well) were transfected into Neuro2a cells using Lipofectamine 2000 Transfection Reagent (Invitrogen, Carlsbd, CA, USA) according to the manufacturer’s protocol. The transfection step was conducted by incubating the cells for 4 h, after which the process was terminated by replacing the transfection medium with DMEM supplemented with 10% FBS. Subsequently, Neuro2a cells were incubated for 24 h. Transfected cells were fixed in 4% paraformaldehyde and permeabilized with 0.25% Triton X-100 in phosphate-buffered saline. Nuclei were stained using 1000 × 6-diamono-2-phenylindole (DAPI; DOJINDO, Kumamoto, Japan), after which the cells were observed by confocal laser scanning microscopy (LSM 710; Carl Zeiss, Oberkochen, Germany). Several fields were randomly selected under 40× magnification using only the DAPI channel that represented a nucleus and more than 100 cells, which appeared as GFP-positive cells with the same level of intensity were counted. The proportion of cells harboring cSOD1 aggregates was estimated. This experiment was conducted five times.

### 4.6. Statistical Analyses

All statistical analyses were conducted using commercial software (JMP 16.0.0 program; SAS Institute, Cary, NC, USA). Data of *T*_m_, ThT fluorescence and the proportion of cells harboring cSOD1 aggregates were analyzed using ANOVA followed by Tukey’s post hoc test for pairwise comparison between groups. Statistical significance was set at *p* < 0.05.

## 5. Conclusions

In this report, we demonstrate the importance of the interaction between amino acid residues E40 and K91 in cSOD1, which stabilize the β-barrel of cSOD1 as the β-barrel plug. The E40K mutation, a major causative mutation of canine DM, is less stable and has a higher amyloidogenic propensity in vitro and in cells. In contrast, the E40K and K91E double mutants recovered this interaction and were stable and less amyloidogenic in vitro and in cells. Interestingly, this combination (K40 and E91) was evolutionarily selected for equine SOD1 and this interaction between amino acid E40 and K91 stabilize the β-barrel of equine SOD1 structure.

## Figures and Tables

**Table 1:** The melting temperature (*T*_m_) of tertiary structure of cSOD1s obtained by DSF.

**Protein Name**	***T*_m_ of the Tertiary Structure Monitored by DSF**
cSOD1(WT)	50.94 ± 0.95
cSOD1(E40K)	46.28 ± 0.51
cSOD1(E40K,K91E)	51.78 ± 0.08

## Data Availability

All datasets obtained and analyzed during the experiment are available upon reasonable request from the respective authors.
